# The PI3K inhibitor copanlisib synergizes with sorafenib to induce cell death in hepatocellular carcinoma

**DOI:** 10.1038/s41420-019-0165-7

**Published:** 2019-04-05

**Authors:** Liangtao Ye, Julia Mayerle, Andreas Ziesch, Florian P. Reiter, Alexander L. Gerbes, Enrico N. De Toni

**Affiliations:** Department of Medicine II, Liver Center Munich, University Hospital, LMU Munich, Munich, Germany

## Abstract

Sorafenib, a multikinase inhibitor targeting the Ras/Raf/MAPK (mitogen-activated protein kinase) and vascular endothelial growth factor signaling pathways is an established treatment option for patients with advanced-stage hepatocellular carcinoma (HCC); however, despite its clinical benefit, chemoresistance and disease progression eventually occur almost invariably during treatment. Activation of the PI3K/AKT (phosphatidylinositol-3-kinase/serine/threonine kinase) pathway plays a role in the pathogenesis of HCC and may contribute to determine resistance to sorafenib. We thus evaluated in vitro the effects of the combination of sorafenib and copanlisib, a PI3K inhibitor recently approved for clinical use. The effects of copanlisib alone and in combination with sorafenib were assessed in several HCC cell lines by proliferation and colony formation assays, fluorescence-activated cell sorting analyses, and western blot. In addition, sorafenib-resistant cell clones were used. Copanlisib strongly reduced cell viability and colony formation in different native and sorafenib-resistant HCC cell lines by affecting cyclin D1/CDK4/6 signaling and causing cell cycle arrest. Elevation of phosphorylated (p)-AKT was observed upon incubation with sorafenib and was consistently found in six different unstimulated sorafenib-resistant cell clones. Copanlisib counteracted sorafenib-induced phosphorylation of p-AKT and synergistically potentiated its antineoplastic effect. In summary, copanlisib shows potent anticancer activity as a single agent and acts synergistically in combination with sorafenib in human HCC. Combination of sorafenib with copanlisib represents a rational potential therapeutic option for advanced HCC.

## Introduction

Hepatocellular carcinoma (HCC) ranks second as cancer-related cause of death and its incidence is expected to increase in the future^1–3^. In the past two decades, the prognosis of HCC has progressively improved owing to the progress of local and locoregional treatment options, the definition of specific criteria for therapeutic stratification and the implementation of screening programs^4,5^. In addition, the recent approval of several new compounds for systemic treatment, such as regorafenib^6^, lenvatinib^7^, nivolumab^8^, and, most recently, cabozantinib^9^, shows that the sequential employment of agents  targeting different signaling pathways is a therapeutic strategy applicable to the treatment of HCC, promising to further ameliorate the life expectancy of patients with advanced-stage tumors. Future treatment schemas will thus likely consist of the combination of different kinase inhibitors simultaneously targeting different intracellular signaling pathways to overcome chemoresistance to single treatment regimens and/or their combination with immune checkpoint inhibitors^10^.

Sorafenib impinges on a number of different signaling pathways, including the Ras/Raf/MAPK (mitogen-activated protein kinase) pathway, Vascular endothelial growth factor (VEGF) and platelet-derived growth factor (PDGF) signaling, hereby affecting different aspects of cell biology, including proliferation, apoptosis, and angiogenesis^11^. However, owing both to the biological heterogeneity of HCC and the broad spectrum of action of sorafenib, in spite of substantial efforts, no reliable predictors of response to this drug could be found yet. Nevertheless, the observation that recurrent molecular changes can be observed in consequence of sorafenib treatment (including increase of c-MET^12^ and the activation of the phosphatidylinositol-3-kinase (PI3K)-serine/threonine kinase (AKT)-mammalian target of rapamycin (mTOR) signaling pathway^13,14^) indicates that inhibition of these signaling pathways could overcome resistance to sorafenib. The compensatory activation of these signaling pathways is in keeping with the significance of c-MET and PI3K-Akt-Tor signaling in the pathogenesis of HCC recently highlighted by extensive genetic investigation of this tumor^15,16^.

Copanlisib (BAY 80-6946) is a recently developed PI3K inhibitor^17–20^, which was lately approved for the treatment of relapsing follicular lymphoma. Here, we aimed at assessing the antineoplastic effect of copanlisib and its potential as a sensitizer to the effect of sorafenib in a preclinical model of HCC.

## Results

### Copanlisib exerts a potent antiproliferative effect as single agent in HCC cells

**Fig. 1:**
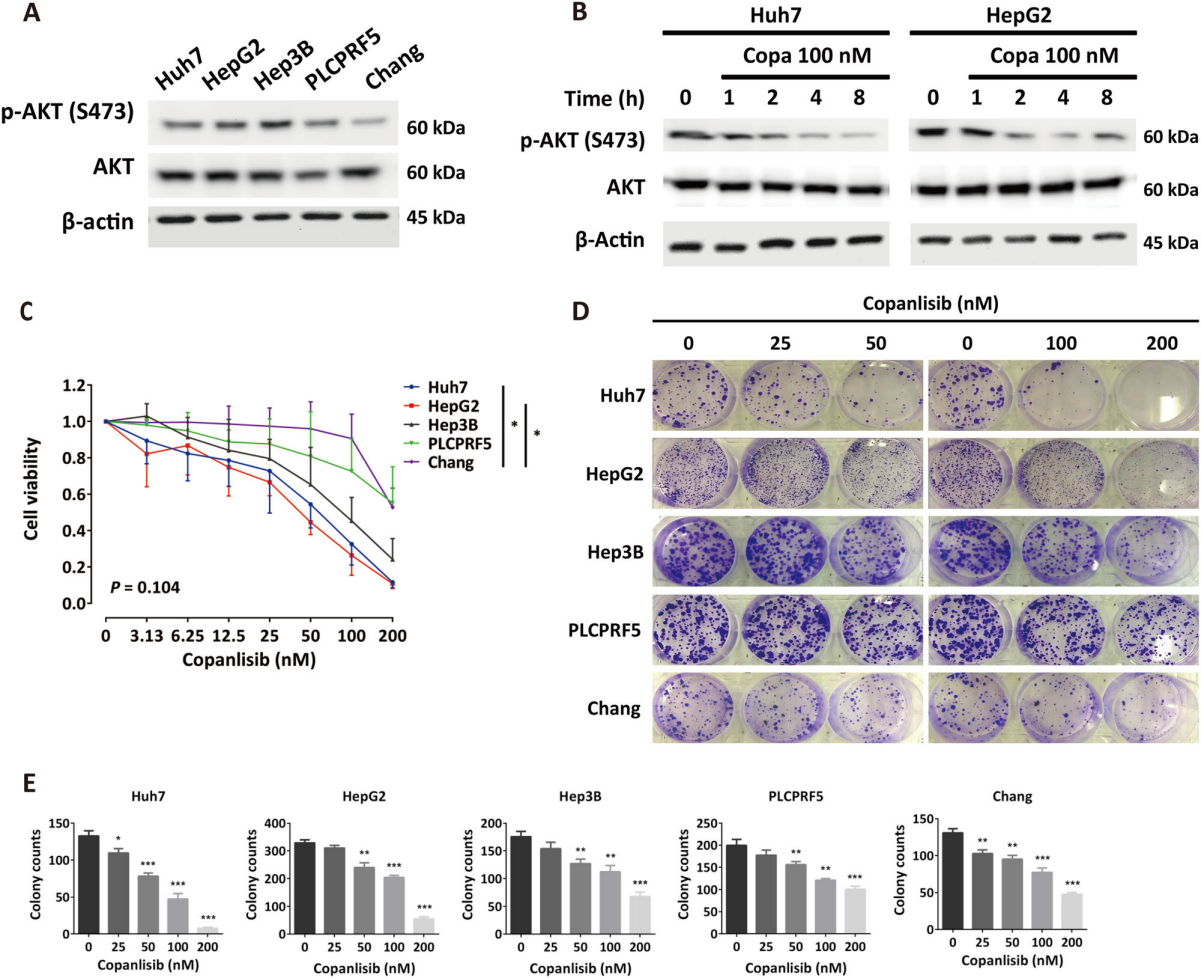
Copanlisib causes reduction of cell viability and colony formation. **a** Baseline levels of phosphorylated (p)-AKT expression in the indicated cell lines. **b** Effect of copanlisib (copa) at the concentration of 100 nM on p-AKT. **c** Changes in cell viability expressed as ratio to control-treated cells at different concentrations of copanlisib. **d** Representative pictures of colony formation of Huh7 and HepG2 cells, and **e** colony count, after incubation with the indicated concentrations. Results are presented as mean and standard deviation (SD) values of at least three experiments. *P < 0.05; **P < 0.01; ***P < 0.001, by *t* test compared with untreated control or analysis of variance (ANOVA) for multiple groups

The effect of copanlisib on cell viability was assessed in five HCC cell lines exhibiting different baseline levels of phosphorylated (p)-AKT (Fig. 1a). As shown in Fig. 1b, the effect of copanlisib on p-AKT was readily observable after 1 h incubation at the concentration of 100 nM. Copanlisib dose-dependently inhibited cell growth in vitro in a clinical concentration range regardless of baseline levels of p-AKT, hereby showing higher potency in Huh7 and HepG2 (half-maximal inhibitory concentration (IC_50_) = 47.9 and 31.6 nM, respectively) vs. Hep3B, PLCPRF5, or Chang cells (IC_50_ = 72.4, 283, and 442 nM, respectively; Fig. 1c). These results were clearly confirmed by clonogenicity assays showing a dose-dependent reduction in the number and size of colonies in Huh7 and HepG2 cells upon incubation with copanlisib (Fig. 1d, e).

### Copanlisib causes G1 cell cycle arrest and down-regulation of cyclin B1 and D1

**Fig. 2:**
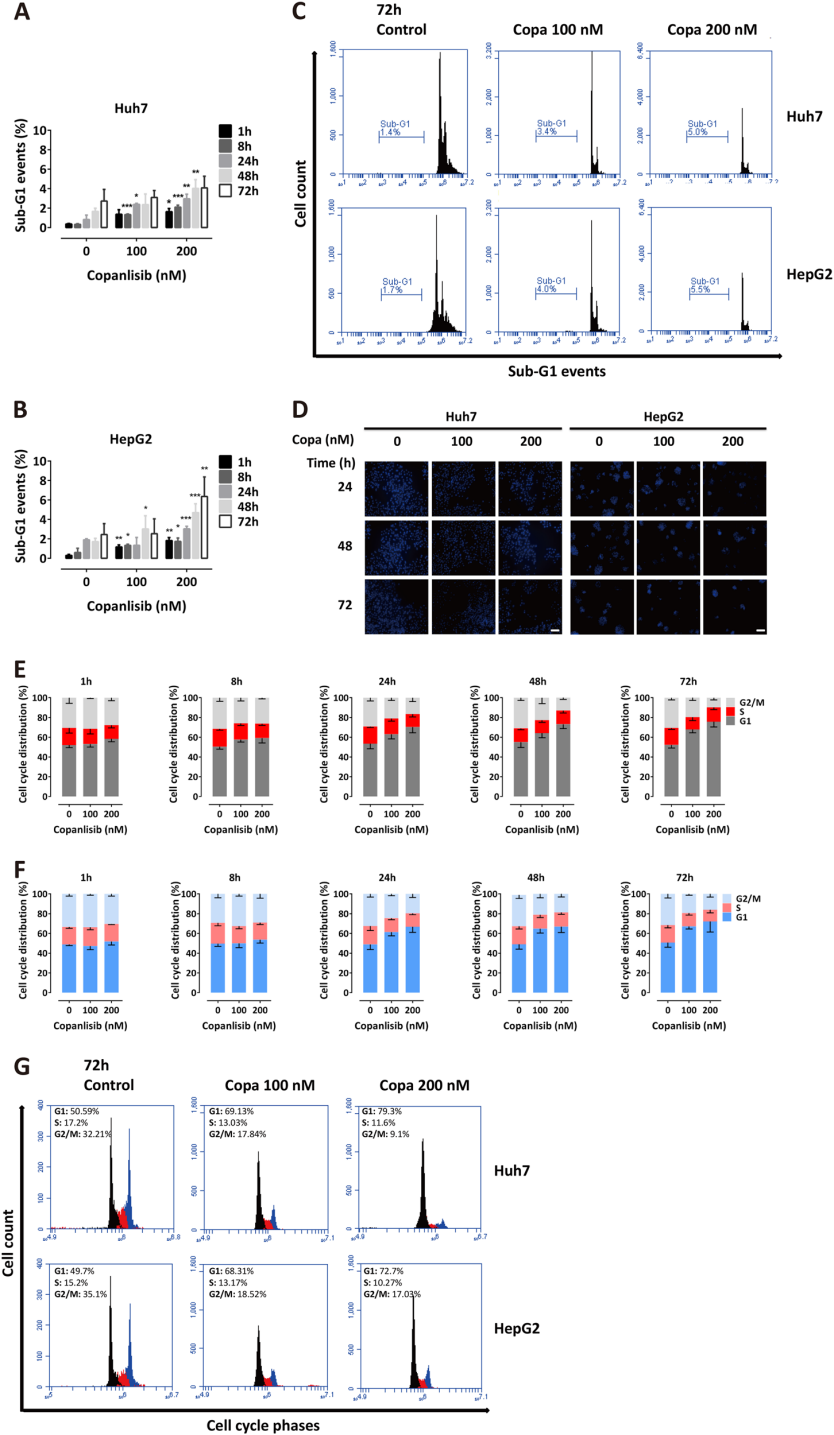
Copanlisib causes a G1 cell cycle arrest. Sub-G1 events upon incubation with copanlisib at the concentrations of 100 and 200 nM in Huh7 (**a**) and HepG2 (**b**) cells showing marginal apoptosis; typical fluorescence-activated cell sorting (FACS) pattern of sub-G1 cell fraction (**c**) and Hoechst staining (**d**) in these cell lines. Cell cycle analysis after incubation with copanlisib (copa) at the indicated concentrations and time points in Huh7 (**e**) and HepG2 (**f**) cells and respective representative features at FACS analysis (**g**). Error bars represent mean ± SD values from at least three experiments. *P < 0.05; **P < 0.01; ***P < 0.001, by *t* test compared with untreated control. In these experiments, fresh medium containing copanlisib at the appropriate concentrations was replaced every day to rule out the possibility that the observed effect might be due to diminishing effect of the agent during incubation

**Fig. 3:**
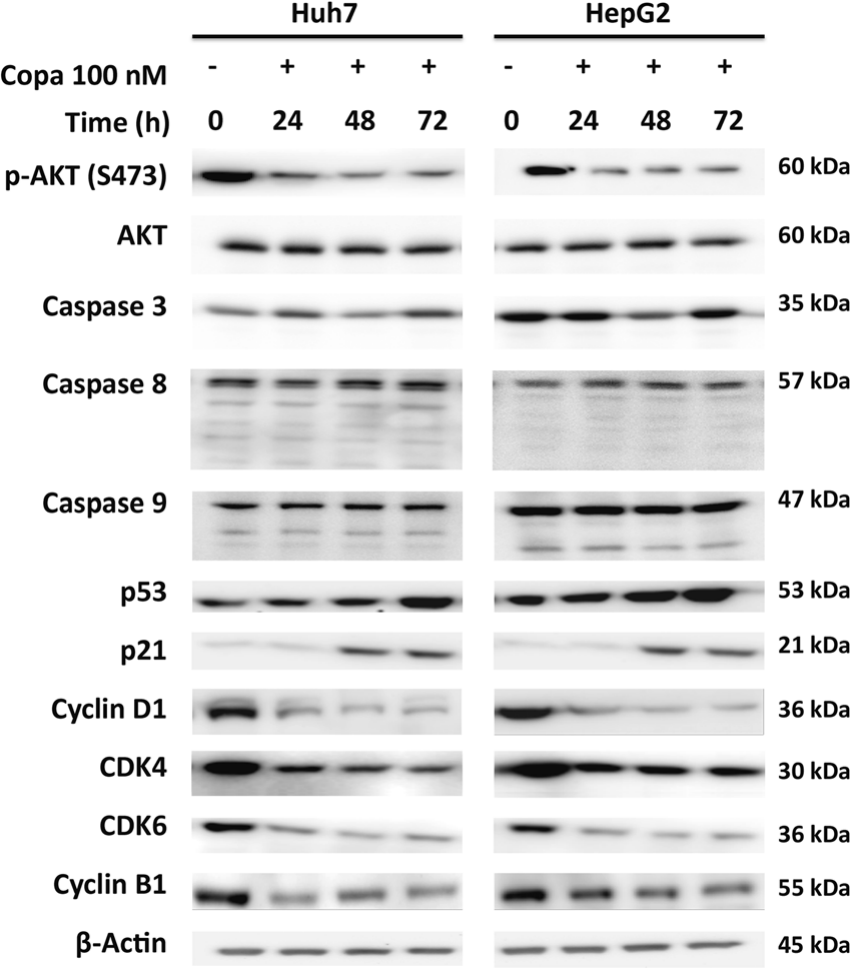
Copanlisib triggers G1 cell cycle arrest by down-regulating cyclin B1 and D1. Immunoblot analyses of different regulators of apoptosis and cell cycle after incubation with copanlisib (copa) at the concentration of 100 nM at the indicated time points. Results are representative of three independent experiments

Since copanlisib was previously shown to cause apoptosis by inhibiting the antiapoptotic protein Bcl-xL in breast cancer cells^21^, apoptosis and cell cycle were assessed to determine their respective contribution to determine the effect of copanlisib in HCC cells. Surprisingly, we found only little or quantitatively negligible apoptosis, as shown by the time kinetic of sub-G1 events at different time points until 72 h after the addition of copanlisib at the concentrations of 100 and 200 nM (Fig. 2a–c). This was confirmed by the absence of typical microscopic features of apoptosis, like chromatin condensation and nuclear fragmentation at Hoechst staining (Fig. 2d). Instead, incubation with copanlisib for up to 72 h caused a clear, dose- and time-dependent G1 cell cycle arrest, along with a corresponding decrease of the S and G2/M phases of cell cycle (Fig. 2e–g). Correspondingly, as key modulators of cell death and cell cycle were assessed, no caspase cleavage could be detected (Fig. 3). Instead, cell cycle arrest was accompanied by a significant reduction of cyclin B1 and cyclin D1 24 to 72 h after the addition of copanlisib. Concomitantly, an increase of both p53 and p21 and a decrease of CDK4 and CDK6 were observed (Fig. 3). These data point to the fact that copanlisib used as a single agent affects the viability of HCC cells mainly by inducing cell cycle arrest by down-regulating CDK4/6 and cyclin D1, which are downstream targets of AKT, but only marginally effects apoptosis.

### Copanlisib synergizes with sorafenib by counteracting sorafenib-induced activation of AKT

**Fig. 4:**
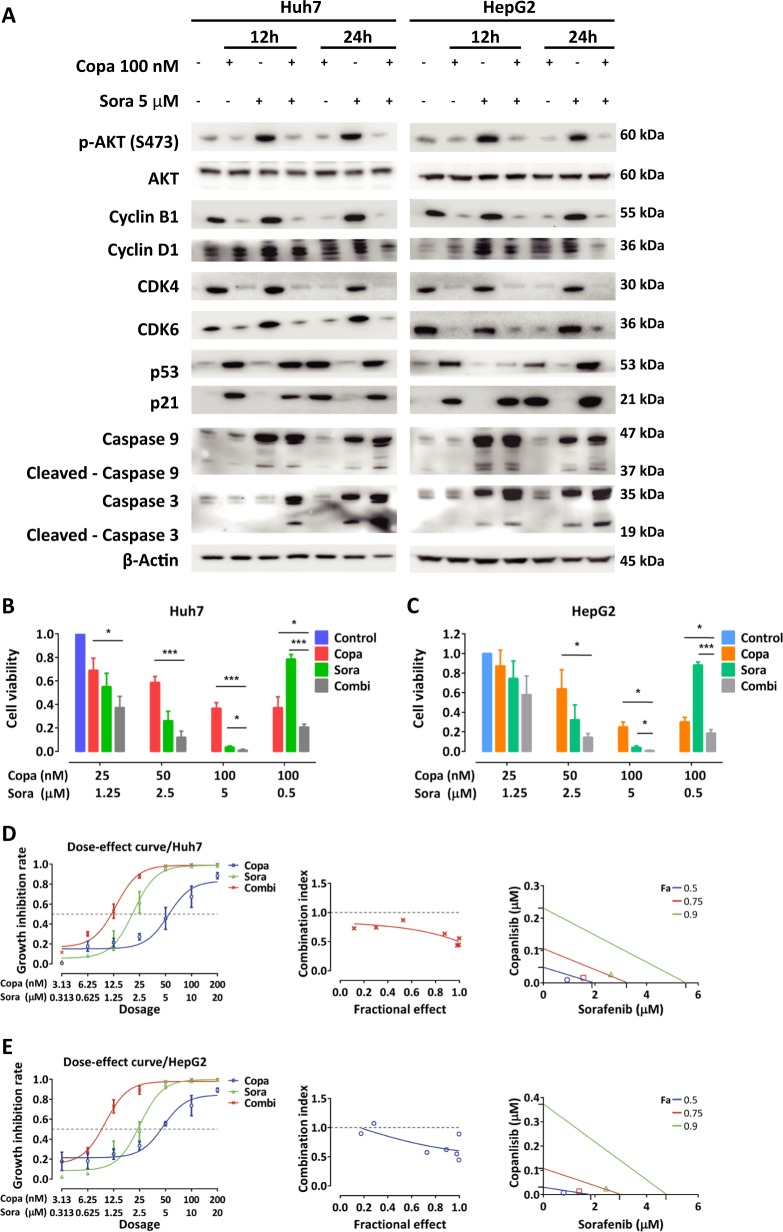
Copanlisib synergizes with sorafenib to determine loss of cell viability by counteracting sorafenib-induced PI3K/AKT (phosphatidylinositol-3-kinase/serine/threonine kinase) activation. **a** Western blot analysis of Huh7 and HepG2 after incubation with copanlisib and/or sorafenib at the indicated concentrations. **b**, **c** Synergistic interaction between sorafenib and copanlisib in Huh7 (**b**) and HepG2 cells (**c**) as determined by assessment of cell viability. Combination index (CI) values <1 indicate a synergistic interaction, a value of 1 indicates additive interaction, and values >1 indicate an antagonistic effect (Copa: copanlisib; Sora: sorafenib; Combi: combination of both substances). **d** Dose–effect relationship of copanlisib, sorafenib, and their combination (constant ratio of 1:100) on growth inhibition of Huh7 cells (left panel). CI values and fraction affected (Fa) for each dose were used to generate the Fa-CI plot (middle panel) and isobologram blots (right panel). Panels in **e** represent for HepG2 the same information provided Huh7 cells. For viability assays, cells were incubated with copanlisib, sorafenib, and their combination for 6 days. Values are expressed as mean ± SD of at least three experiments. *P < 0.05; **P < 0.01; ***P < 0.001, by *t* test between two groups

Since sorafenib-induced phosphorylation of AKT is thought to represent a mechanism counteracting the antineoplastic effect of sorafenib^14,22,23^, we assessed the effect of combined sorafenib and copanlisib in HCC cells. As expected, sorafenib strongly diminished cell viability alone hereby causing increased expression and cleavage of procaspase-3 and -9 (Fig. 4a). Concomitantly however, sorafenib induced increase of p-AKT, cyclin B1, and cyclin D1 an effect which could be reversed by co-incubation with copanlisib. In addition, co-administration of sorafenib and copanlisib increased caspase cleavage. The antineoplastic interaction between sorafenib and copanlisib was confirmed by combination index (CI) and isobologram blots showing that these agents synergize to determine the loss of cell viability in Huh7 and HepG2 cells (Fig. 4b–e). Altogether, these data suggest that sorafenib-mediated activation of AKT and cyclin D1 might represent a mechanism of resistance limiting the antineoplastic potential of sorafenib.

**Fig. 5:**
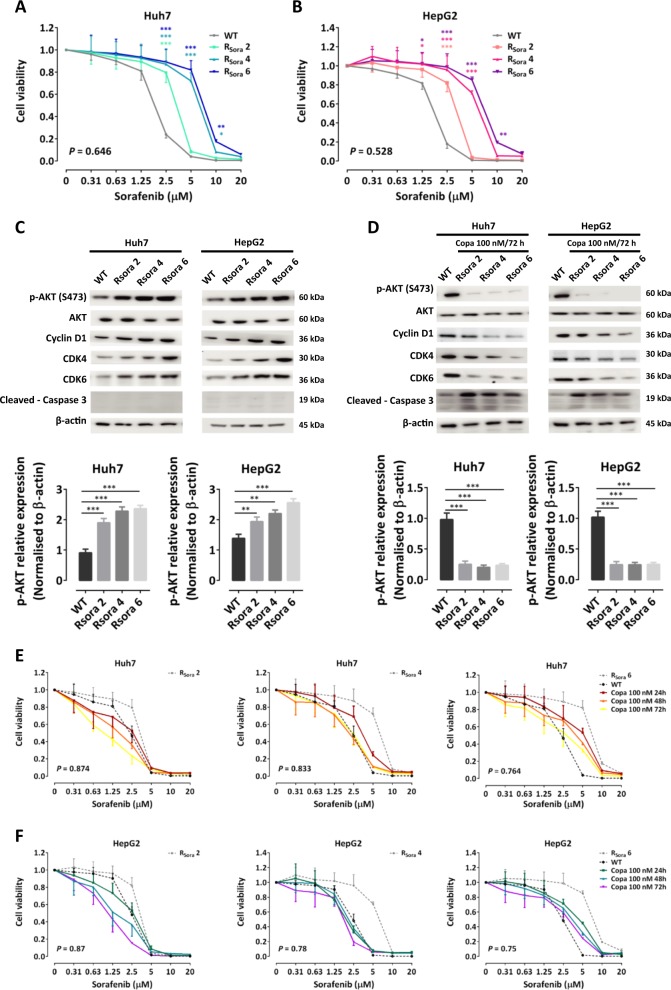
Copanlisib restores the sensitivity of sorafenib-resistant Huh7 and HepG2 cell lines to sorafenib. **a**, **b** Viability assay after incubation with sorafenib in wild-type (WT) Huh7 and HepG2 cells and in sorafenib-resistant cells at the respective concentrations of 2 µM (Rsora 2), 4 µM (Rsora 4), and 6 µM (Rsora 6). **c** Western blot of phosphorylated (p)-AKT and AKT in unstimulated Huh7 and HepG2 (WT) and in the indicated sorafenib-resistant cell lines. **d** Western blot of p-AKT in sorafenib-resistant Huh7 and HepG2 cells after incubation to copanlisib for 72 h. **e**, **f** Cell viability assays showing the effect of combined administration of copanlisib and sorafenib in the indicated sorafenib-resistant Huh7 (**e**) or HepG2 (**f**) cells. The results are shown as mean ± and standard deviation (SD) of three experiments. *P < 0.05; **P < 0.01; ***P < 0.001, by *t* test between two groups or analysis of variance (ANOVA) for multiple groups

To confirm these findings, we established several cell lines with acquired resistance to sorafenib by long-term exposure to increasing concentrations of this agent. As expected, such Huh7- and HepG2-derived cells lines were less sensitive to sorafenib in comparison to their wild-type counterpart (Fig. 5a, b). In addition, they exhibited increased levels of p-AKT, cyclin D1, CDK4, and CDK6 (Fig. 5c), an effect that could be counteracted by copanlisib (Fig. 5d). Correspondingly, as shown by viability experiments, copanlisib restored sorafenib sensitivity in sorafenib-resistant cells (Fig. 5e–f).

Taken together, these results indicate that increase of AKT is not only triggered in response to sorafenib but also represents a mechanism of acquired resistance developing after long-term exposure to this agent. AKT inhibition by copanlisib counteracts this mechanism of resistance hereby sensitizing cancer cells to sorafenib.

### Co-treatment with copanlisib and sorafenib enhances apoptotic cell death in a caspase-dependent way

**Fig. 6:**
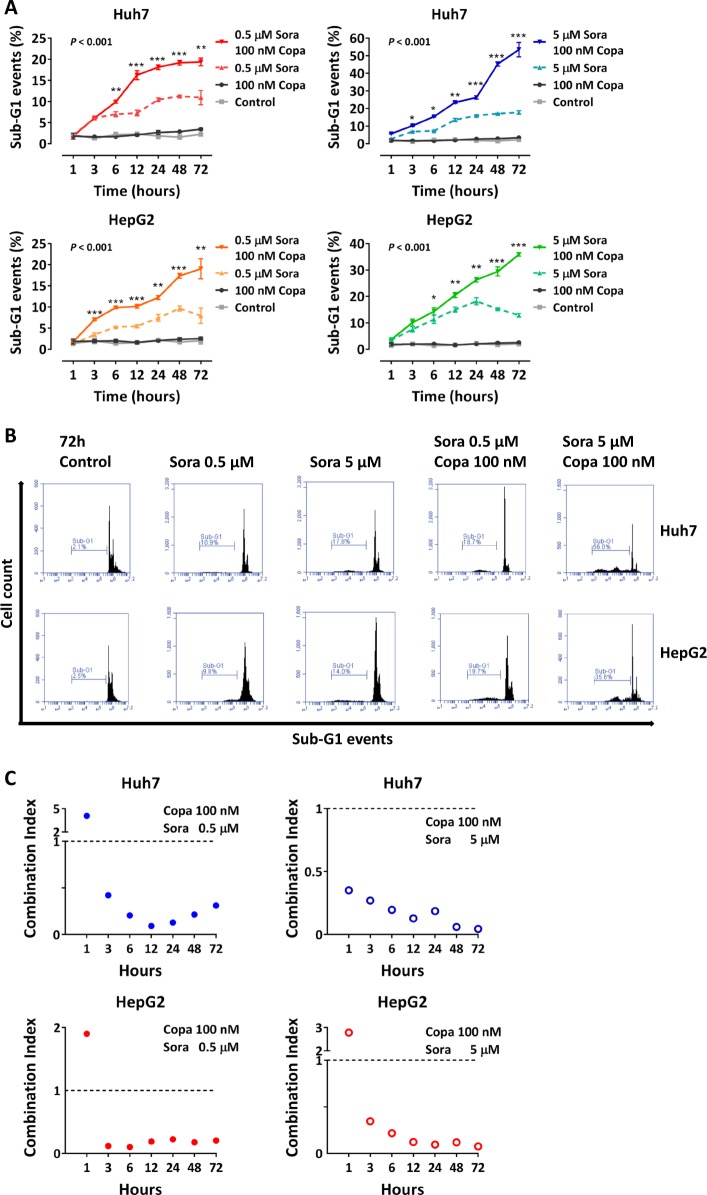
Copanlisib sensitizes hepatocellular carcinoma (HCC) cells to sorafenib by increasing susceptibility to apoptosis. **a** Sub-G1 events and typical fluorescence-activated cell sorting (FACS) patterns (**b**) after incubation with copanlisib with or without the addition of increasing concentrations of sorafenib in Huh7 and HepG2 cell lines. **c** Combination index (CI) plot of sub-G1 events showing a synergistic interaction of sorafenib and copanlisib in determining apoptosis. Results are represented as mean ± SD of three expriments. *P < 0.05; **P < 0.01; ***P < 0.001, by *t* test between two groups or analysis of variance (ANOVA) for multiple groups (Copa, copanlisib; Sora, sorafenib)

**Fig. 7:**
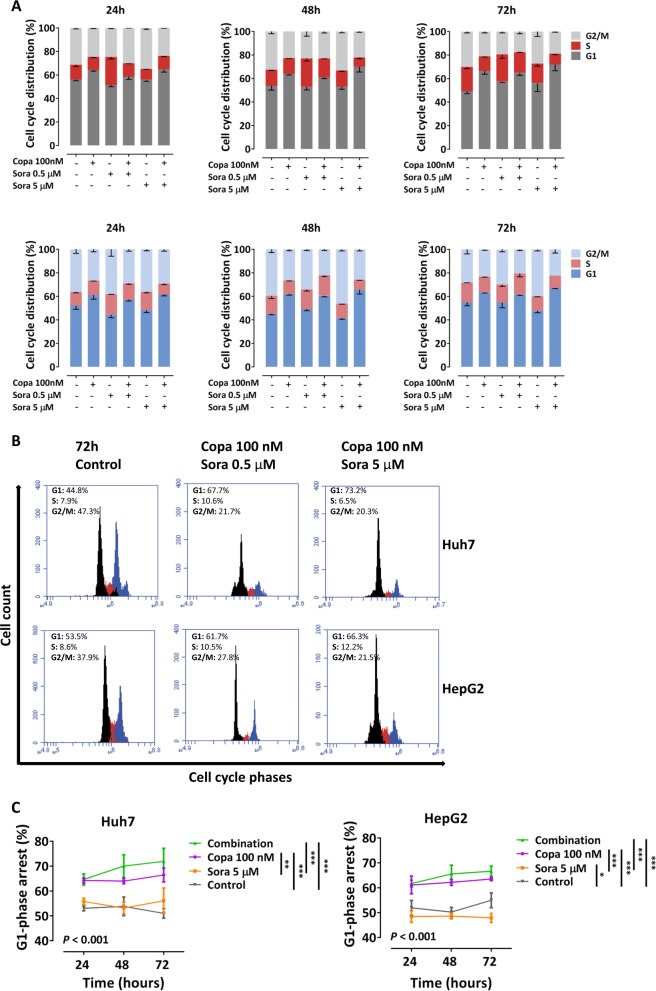
Copanlisib sensitizes hepatocellular carcinoma (HCC) cells to sorafenib. **a** Cell cycle analysis and typical representative fluorescence-activated cell sorting (FACS) analysis diagrams (**b**) of cells incubated with copanlisib and sorafenib. **c** Time cause of the G1 phase. Diagrams show mean and standard deviation (SD) of three experiments. *P < 0.05; **P < 0.01; ***P < 0.001, by analysis of variance (ANOVA) test (Copa, copanlisib; Sora, sorafenib)

**Fig. 8:**
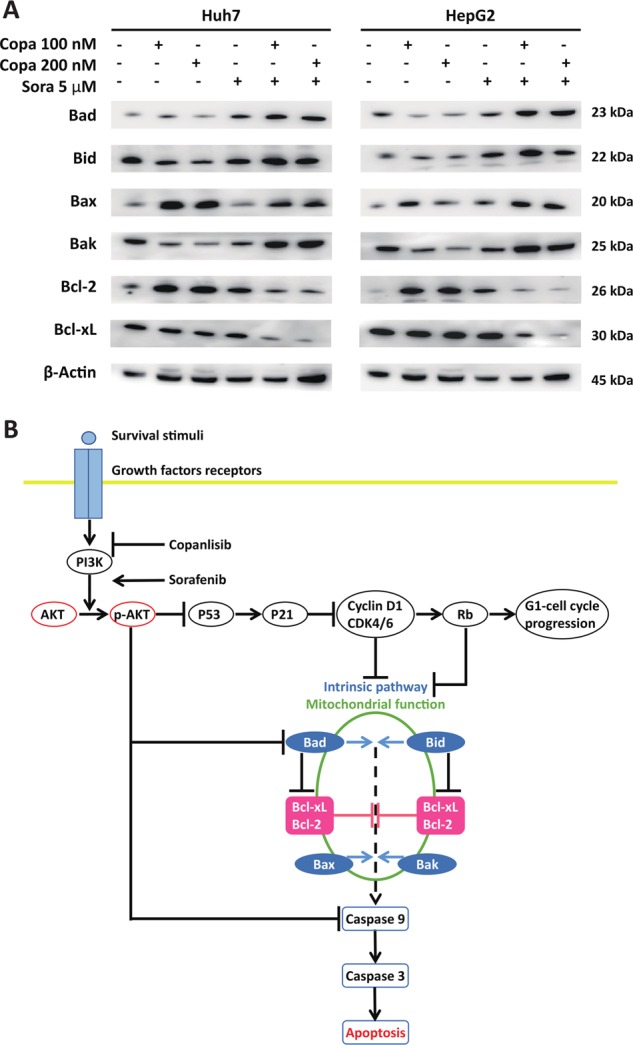
Effects of copanlisib and sorafenib on different intracellular signaling pathways in hepatocellular carcinoma (HCC) cells. **a** Western blot of critical regulators of apoptosis after stimulation with copanslisib and/or sorafenib for 72 h. Data are representative of three independent repeats (Copa, copanlisib; Sora, sorafenib). **b** Schematic diagram describing the hypothesized effect of copanlisib, sorafenib, and their interaction on cell cycle and apoptosis

To investigate the mechanisms underlying the synergistic interaction between copanlisib and sorafenib in Huh7 and HepG2 cells, we examined the effect of their combination on cell cycle and apoptosis. In agreement with the observed increase of caspase cleavage caused by co-stimulation with both agents (Fig. 4a), a synergistic increase of apoptosis (Fig. 6a–c) and an additive increase of cell cycle arrest were observed (Fig. 7a–c). At the molecular level, western blot analysis showed that the combined substances cause an increase of the proapoptotic proteins Bad, Bid, Bax, and Bak and a decrease of the antiapoptotic molecules Bcl-2 and Bcl-xL (Fig. 8a).

## Discussion

Derangement of the PI3K signaling pathway is one of the most common events in the pathogenesis of cancer ^24,25^ and is thought to play a role in the development of ~50% of HCC^15^. Sorafenib, a standard first-line treatment option for HCC, is known to affect the activity of a broad spectrum of kinases, but has little effect on the activity of the PI3K-Akt-Tor signaling, a pathway that, as recently proposed^26,27^, might represent a mechanism of acquired resistance to this agent.

Copanlisib is a newly developed pan-PI3K inhibitor that has most recently been approved for the treatment of relapsed follicular lymphoma^28^ and is undergoing clinical experimentation for the treatment of indolent or aggressive non-Hodgkin lymphoma^29^. In addition, copanlisib is being assessed for the treatment of solid tumors in preclinical studies^17,20,21^ and in early-phase clinical trials, showing signs of effectiveness and acceptable toxicity^30,31^. The availability of this agent for clinical use represented the rationale for assessing its combined effect with sorafenib for the treatment of HCC.

In our model, copanlisib showed remarkable antiproliferative properties in different HCC cell lines independently of their basal expression of AKT. Previous investigation in breast cancer cells had shown that copanlisib causes apoptosis by down-regulating the antiapoptotic protein Bcl-xL^21^. In contrast, our findings show that copanlisib suppresses tumor growth in HCC cells mainly by triggering a G1 cell cycle arrest by inhibiting the cyclin D1/CDK4/CDK6 axis (Fig. 8b), without causing apoptosis (Fig. 3). Cyclin D1, CDK4, and CDK6 are regulated by the PI3K-Akt-Tor signaling pathway and control the progression from the G1 to the G2 phase of cell cycle by phosphorylating the tumor suppressor Rb, which in turn causes the activation of the transcription factor E2F^32^. Extensive efforts to characterize the mutational landscape of HCC by exome sequencing recently conducted by the group of Zuckmann-Rossi and colleagues^15^ has revealed that amplification of cyclin D1 is one of the most frequent events during HCC development and is independently associated with a poor prognosis. The frequent activation of the PI3K-Akt-Tor signaling and amplification of cyclin D1 in HCC corroborate the rationale for assessing copanlisib as a treatment option in patients with advanced HCC.

In addition to its antiproliferative properties as single agent, copanlisib also synergistically potentiated the effect of sorafenib, which is consistent with the notion that the activation of PI3K-Akt-Tor signalling serves as mechanism of escape from the inhibition of the Ras-Raf-MAPK axis. We correspondingly found that p-AKT as well as cyclin B1, cyclin D1, and CDK4/6 were increased by sorafenib, an effect that could be completely abrogated by copanlisib. Our observations are in accordance with previous studies showing that interruption of PI3K/AKT signaling sensitizes tumor cells to sorafenib-induced apoptosis^14^ and with the fact that we consistently found high basal levels of p-AKT, CDK4/6, and cyclin D1 in cell lines with acquired resistance to sorafenib.

Interestingly, we observed that the mechanisms underlying the effects of the combined agents were different from those observed with copanlisib alone. Copanlisib had little effect on apoptosis, which was instead remarkably increased by the combination of both agents. This proapoptotic effect was caused by the activation of the intrinsic signaling pathway, in particular owing to the profound down-regulation of the antiapoptotic molecules Bcl-2 and Bcl-xL, an effect that was previously suggested as a mechanism of action of copanlisib in breast cancer cells^21^.

While our results suggest the possibility that copanlisib could be used as treatment option for patients with advanced HCC alone or in combination with sorafenib, copanlisib might also be used in combination with other anticancer agents, comprising immune checkpoint inhibitors. The association of immune checkpoint inhibitors with kinase inhibitors seems to represent a promising therapeutic approach, as exemplified by a recent early-stage trial with combined lenvatinib and pembrolizumab^33^. Since clinical evidence was provided on the efficacy of combined mTOR inhibitors and PD-1/PD-L1-blocking agents in HCC and metastatic renal cell carcinoma^34,35^, copanlisib could be used in combination with immune checkpoint inhibitors to potentiate the effect of these agents.

## Conclusion

In summary, we highlight the role of PI3K-Akt-Tor signaling activation in inducing resistance to sorafenib, and shed light on the mechanism of action underlying the antineoplastic effects of copanlisib and its synergistic interaction with sorafenib. We suggest that copanlisib is considered as a potential treatment option for HCC alone or in combination with sorafenib.

## Materials and methods

### Cell lines and reagents

Huh7, HepG2, PLCPRF5, and Chang cells were cultured in Dulbecco’s modified Eagle’s medium and Hep3B cells were cultured in Eagle’s minimum essential medium (Sigma-Aldrich, Munich, Germany) with 10% fetal bovine serum (Biochrom GmbH, Berlin, Germany) and 1% penicillin–streptomycin (Sigma-Aldrich, Munich, Germany). Cells were maintained in 5% CO_2_ at 37 °C. Copanlisib (BAY 80-6946, Selleckchem, Munich, Germany) and sorafenib (Selleckchem, Munich, Germany) were stored at −20 °C and dissolved in 100% dimethyl sulfoxide at a stock concentration of 5 and 43 mmol, respectively. Two sorafenib-resistant sublines were established from Huh7 and HepG2 cells, termed sorafenib-resistant-Huh7 (Huh7-SR) and sorafenib-resistant-HepG2 (HepG2-SR), by incubation with increasing doses of sorafenib (2, 4, and 6 µM) for at least 8 months. Authentication of cell lines was conducted by the Leibniz Institute DSMZ-German Collection of Microorganisms and Cell Cultures.

### Cell proliferation assays

One thousand to 1500 cells were seeded in 96-well plates, cultured overnight, and then incubated in the presence of various concentrations of copanlisib and/or sorafenib. After 6 days, cells were washed with phosphate-buffered saline and underwent osmotic lysis in 100 µl ddH_2_O for 45 min at 37 °C. Zero point two percent Sybr green (Lonza, Rockland, ME, USA) was added to each well, fluorescence was measured (GloMax-Multi + Detection System with Instinct Software, Promega, USA) and proliferation index was calculated as a ratio to untreated samples. Three independent experiments were performed per agent, with each data point reflecting triplicate wells. Error bars represented standard deviation of the mean (SD) from three experiments. Data were analyzed by the median-effect method (CompuSyn software; Biosoft, Ferhuson, MO, USA) to determine the drug concentrations resulting in 50% growth inhibition (IC_50_). The isobologram method and Chou–Talalay CI, a well-established index reflecting the interaction of two drugs, was calculated at different levels of growth inhibition with the use of CompuSyn software^36^. CI values of <1, 1, and >1 indicated synergistic, additive, and antagonistic effects, respectively.

### Colony formation assay

Five thousand cells were seeded in 6-wells plates. After overnight incubation, cells were exposed to copanlisib for 48 h. Fresh medium was replaced regularly and the plates were maintained at 37 °C for 3 weeks. Colonies were counted after fixation in 9% paraformaldehyde and staining with crystal violet for 30 min (Sigma, St. Louis, MO, USA).

### Apoptosis and cell cycle assays

Cells (8.0 × 10^4^ to 1.5 × 10^5^) were seeded in 6-well plates and, after overnight incubation, treated with the indicated agents. After propidium iodide staining, fluorescence-activated cell sorting (Accuri C6 flow cytometer, BD Biosciences San Jose, CA, USA) was performed. Apoptosis was quantified by measuring the fraction of cells with sub-diploid DNA content (sub-G1) and assessed morphologically by Hoechst staining and fluorescence microscopy (Zeiss, Jena, Germany).

### Western blot

Equal amount of proteins in each sample was loaded on 10/12% sodium dodecyl sulfate-polyacrylamide gel electrophoresis gels and separated for 25 min at 80 V and for 80 min at 120 V, and then transferred onto PVDF membranes (EMD Millipore Corporation, Billerica, MA, USA). After blocking for 1 h in TBST (Tris-buffered saline with Tween-20) containing 5% milk or 5% bovine serum albumin, membranes were incubated overnight at 4 °C with the following primary antibodies: p-AKT (S473), AKT, β-actin, caspase-3, caspase-8, caspase-9, cyclin B1, cyclin D1, p53, p21, CDK4, CDK6, Bad, Bax, Bak, Bcl-2, Bcl-xL (Cell Signaling Technology, USA), and Bid (FL-195; Santa Cruz, Germany). Subsequently, the membrane was probed with horseradish-peroxidase-conjugated secondary anti-rabbit or -mouse immunoglobulin G antibodies (GE Healthcare UK Limited) for 2 h at room temperature at concentrations of 1:10,000. The bands were visualized by SuperSignal West Pico Chemiluminescent Substrate (Thermo Scientific, Schwerte, Germany) and photographed with an image acquisition system (ECL ChemoCam Imager, Intas GmbH, Germany).

### Statistical analysis

Data were analyzed by SPSS version 22.0 (SPSS Inc., IL USA). Results were shown as mean ± standard deviation of at least three independent experiments. Statistical difference were assessed by Student’s *t* test between two groups and one-way analysis of variance test followed by the least significant difference method for multiple groups. Statistical significance was defined by *P* values <0.05; *P < 0.05; **P < 0.01; ***P < 0.001. The CI was determined as described above^36^.
